# The Altered Pattern of the Functional Connectome Related to Pathological Biomarkers in Individuals for Autism Spectrum Disorder Identification

**DOI:** 10.3389/fnins.2022.913377

**Published:** 2022-05-06

**Authors:** Liling Peng, Xiao Liu, Di Ma, Xiaofeng Chen, Xiaowen Xu, Xin Gao

**Affiliations:** ^1^Shanghai Universal Medical Imaging Diagnostic Center, Shanghai, China; ^2^School of Business Administration, José Rizal University, Mandaluyong, Philippines; ^3^College of Information Science and Technology, Nanjing Forestry University, Nanjing, China; ^4^College of Mathematics and Statistics, Chongqing Jiaotong University, Chongqing, China; ^5^Department of Medical Imaging, Tongji Hospital, Tongji University School of Medicine, Tongji University, Shanghai, China

**Keywords:** Pearson’s correlation, functional magnetic resonance imaging, functional brain network, autism spectrum disorder, MK-SVM

## Abstract

**Objective:**

Autism spectrum disorder (ASD) is a common neurodevelopmental disorder characterized by the development of multiple symptoms, with incidences rapidly increasing worldwide. An important step in the early diagnosis of ASD is to identify informative biomarkers. Currently, the use of functional brain network (FBN) is deemed important for extracting data on brain imaging biomarkers. Unfortunately, most existing studies have reported the utilization of the information from the connection to train the classifier; such an approach ignores the topological information and, in turn, limits its performance. Thus, effective utilization of the FBN provides insights for improving the diagnostic performance.

**Methods:**

We propose the combination of the information derived from both FBN and its corresponding graph theory measurements to identify and distinguish ASD from normal controls (NCs). Specifically, a multi-kernel support vector machine (MK-SVM) was used to combine multiple types of information.

**Results:**

The experimental results illustrate that the combination of information from multiple connectome features (i.e., functional connections and graph measurements) can provide a superior identification performance with an area under the receiver operating characteristic curve (ROC) of 0.9191 and an accuracy of 82.60%. Furthermore, the graph theoretical analysis illustrates that the significant nodal graph measurements and consensus connections exists mostly in the salience network (SN), default mode network (DMN), attention network, frontoparietal network, and social network.

**Conclusion:**

This work provides insights into potential neuroimaging biomarkers that may be used for the diagnosis of ASD and offers a new perspective for the exploration of the brain pathophysiology of ASD through machine learning.

## Introduction

As a neural developmental syndrome, autism spectrum disorder (ASD) is commonly defined as defective or restricted communication, repetitive behaviors, and social reciprocity, leading to dysfunction in social, educational, and professional fields (Lord et al., 2000; Frith and Happé, 2005; Baio, 2014; Wee et al., 2016); the definition is based on the diagnosis and disease severity assessment. Approximately 1.47% of American children present with some form of ASD, the incidence of which has increased by nearly 30% per 2 years (Baio, 2014). Unfortunately, the diagnosis of ASD is dependent on the symptoms and behavioral patterns of ASD (Gillberg, 1993; Segal, 2000; Lord and Jones, 2012), due to which timely and appropriate treatments cannot be availed. Meanwhile, although gene expression-based diagnostic methods (Wang et al., 2009; O’Roak et al., 2012) can benefit early diagnosis, they are often costly and complicated. Fortunately, several studies have illustrated that abnormal functional disruptions in certain brain regions (Allen and Courchesne, 2003; Anderson et al., 2011; Delmonte et al., 2012) are highly correlated with ASD. Therefore, a potential method of identifying biomarkers for ASD can be adopted by analyzing brain activity data.

Functional magnetic resonance imaging (fMRI) has been successfully utilized in brain mechanism research and clinical diagnosis (Brunetti et al., 2006; Kevin et al., 2008; Jin et al., 2010; Li et al., 2020b). Particularly, several studies have suggested that patients with ASD show atrophy of gray matter volume, degeneration of white matter fiber structure, and reduction of spontaneous functional activity in the hippocampus and frontal region (Brambilla et al., 2003; Anderson et al., 2011; Li et al., 2017). However, because spontaneous brain activities are asynchronous and random across subjects, direct comparison of fMRI data (i.e., time courses) to identify and distinguish ASD patients from normal controls (NCs) remains challenging. As an alternative, the functional brain network (FBN) can provide insights into an effective method that can be developed to obtain data on relatively stable biomarkers that can be used for ASD identification (Smith et al., 2011; Sporns, 2011; Wee et al., 2012; Stam, 2014; Rosa et al., 2015). Moreover, several studies have confirmed that significant changes in FBN are highly correlated with neurological diseases such as ASD (Theije et al., 2011; Gotts et al., 2012), Alzheimer’s disease (Supekar et al., 2008; Huang et al., 2009; Liu et al., 2012), and mild cognitive impairment (Gao et al., 2020), among others.

Specifically, brain connectome analysis, including functional connections and graph theory topological measurements, has attracted significant attention owing to the complex brain network mechanism and various types of diagnostic information (Biswal et al., 1995; Li et al., 2019, 2021). In a series of resting-state FBN studies, several attempts have been reported on the consideration of the functional connections among individuals to train a classifier and findings have revealed a significant change in the default mode network (DMN), salience network (SN), and language network (O’Roak et al., 2012; Nielsen et al., 2013; Verly et al., 2014; Wee et al., 2014; Li et al., 2017). Other ASD studies have focused on utilizing graph theory to reveal the difference (Keown et al., 2017). However, most studies were conducted separately depending on brain network functional connections, and a certain portion of the information derived from the graph theory attributes might have been lost, which limited their performance. Thus, a more robust tactic is to combine the information of the brain network functional connections and graph theory attributes. Considering the potential superiority of multi-view learning tricks, we utilized the most commonly considered multi-kernel support vector machine (MK-SVM) method to combine information for accurate ASD diagnosis. This study may provide valuable insights into the pathophysiological mechanisms of preclinical ASD. The main contributions of this study can be summarized as follows.

1) Our findings revealed a new pathway that could be considered to efficiently identify and distinguish ASD from NCs by combining information from the brain network functional connections and graph theory attributes.
2) We used the MK-SVM as an example to confirm the information combination approach by identifying the ASD from NCs and achieved an 82.60% classification accuracy, which demonstrated a competitive finding.
3) A graph theoretic analysis suggested that the discriminative brain regions of ASD patients were mainly distributed in the limbic system, subcutaneous nuclei, cortex, and connections among them, which corresponded to the SN, DMN, attention network, frontoparietal network, and social network.
4) Our research further revealed that ASD patients showed enhanced integration function and weakened segregation functions of the brain network. Additionally, the functional connections related to the medial temporal lobe (e.g., the parahippocampal gyrus, hippocampus, and entorhinal cortex) and subcutaneous nuclei (e.g., putamen and pallidum) were mainly increased, while the related connections of the frontal lobe, parietal lobe, and occipital lobe were mainly decreased.

## Materials and Methods

### Data Acquisition

We collected the resting-state fMRI (rs-fMRI) data related to 47 NC subjects and 45 ASD subjects (with ages ranging from 7 to 15 years), and data were deduced from a publicly available dataset named ABIDE (Di et al., 2014). There were no significant differences in gender and age between the ASD and NC groups, and the demographic information of the samples has been listed in Table 1. Data were similar to those reported in a recent study (Wee et al., 2016). For more details, refer to Wee et al. (2016).

**TABLE 1:** Demographic information of the samplings.

	*ASD*(*N* = 45)	*NC*(*N* = 47)	*p*−Value
Gender(M/F)	36/9	36/11	0.2135*
Age(*year*±*SD*)	11.1 ± 2.3	11.0 ± 2.3	0.7773^†^
FIQ(*mean*±*SD*)	106.8 ± 17.4	13.3 ± 14.1	0.0510
*ADI*−R(*mean*±*SD*)	32.2 ± 14.3^‡^	–	–
*DOS*(*mean*±*SD*)	13.7 ± 5.0	–	–

ADI-R, Autism Diagnostic Interview-Revised; FIQ, Full Intelligence Quotient; ADOS, Autism Diagnostic Observation Schedule; ASD, autism spectrum disorders; NC, normal control. *The p-value was obtained by chi-squared test. ^†^The p-value was obtained by two-sample two-tailed t-test. ^‡^Two patients do not have the ADI-R score.

### Data Preprocessing

All rs-fMRI images were acquired using the 3T Siemens Allegra scanner. The imaging parameters included flip angle = 90°, TR/TE = 2000/15ms with 180 volumes, 33 slices, and 4.0mm voxel thickness. Specifically, the SPM8 toolbox^1^ and DPARSFA (version 2.2)^2^ were adopted to execute the fMRI pre-processing pipeline. Particularly, the pre-processing pipeline in this study is referenced to the well-defined pipeline reference in the DPABI manual, including (1) the removal of the first 10 time series; (2) normalization; (3) regression of nuisance signals (ventricle, white matter, global signals, and head motion) with the Friston 24-parameter model (Friston et al., 1996); (4) filtering of data (0.01−0.08Hz); and (5) the conduction of de-trending. Subsequently, the pre-processed BOLD time-series signals were partitioned into 90 ROIs according to the automated anatomical labeling atlas (Tzourio-Mazoyer et al., 2002).

### Network Estimation

To define the network, we adopted the Pearson correlation (PC) to estimate FBNs, the details of which have been expressed as follows:


(1) $$W_{i\,j}=\frac{{\left(x_{i}-{\bar{x}}_{i}\right)}^{T}\,\left(x_{j}-{\bar{x}}_{j}\right)}{\sqrt{{\left(x_{i}-{\bar{x}}_{i}\right)}^{T}\,\left(x_{i}-{\bar{x}}_{i}\right)}\,\sqrt{{\left(x_{j}-{\bar{x}}_{j}\right)}^{T}\,\left(x_{j}-{\bar{x}}_{j}\right)}}$$

where *x*_*i*_ ∈ *R^t^*represent the average BOLD signal corresponding to the *i*-th brain region, *t* represents the time length, ${\bar{x}}_{i}\in R^{t}$ represents a vector whose elements are the mean values of the elements in *x*_*i*_, *i* = 1,2,⋯,*n*, and *n* represents the number of ROIs.

### Computation of Graph Measurements

To investigate the altered reconfiguration patterns of individual brain connectomes for ASD, we first performed a graph theory analysis of the FBN based on the graph network analysis toolbox (Gretna).^3^ Specifically, we considered both global graph theory measurements and the nodal property to characterize the different patterns of connections in the brain network, as shown in Table 2. The definitions of these measurements can be found in the paper published by Wang et al. (2015). Note that we focused on the binary network by considering the different sparsity thresholds (ranging from 0.02 to 0.5, with steps of 0.01). A total of 49 values under the sparsity threshold were obtained for each graph measurement. We then utilized the area under the curve (AUC; the sum of 49 values) as input for the attributes to train the classifier, which ensure that there was only one for each node value corresponding to one graph metric.

**TABLE 2:** Selected global and local graph measurements.

Global graph measurements	Local graph measurements
Characteristic path length (*L*_*p*_)	Degree centrality
Clustering coefficient (*C*_*p*_)	Nodal efficiency
Normalized characteristic path length (λ)	Betweenness centrality
Normalized clustering coefficient (γ)	Shortest path length
Small-world(σ)	Nodal clustering coefficient
Global efficiency (*E*_*global*_)	
Local efficiency (*E*_*local*_)	
Modularity score (*Q*)	
Assortativity (*Ar*)	
Hierarchy (*Hr*)	
Synchronization (*Sr*)	

### Hub Node of the Estimates Functional Brain Network

The top 5% regions of the brain with the greatest weight were selected as the hubs of the group-level brain network. Specifically, we utilized the mean value of the entire individual brain network to establish the ASD/HC group-level network.

### Information Combination for Autism Spectrum Disorder Identification

To accurately identify ASD from NCs, we attempted to combine the information from the connection weight and its topological information. We used the multi-kernel (MK) trick to combine different types of information. Specifically, we utilized the LIB-SVM toolbox to solve the support vector machine (SVM) classification problem. The MK-SVM is used to solve the following primary problem:


(2) $$\begin{array}{c} \min_{\text{W}}\frac{1}{2}\,\sum_{m=1}^{3}\beta_{m}\,{||w^{m}||}^{2}+C\,\sum_{i=1}^{\text{n}}\xi_{i}\\ \text{s}.\text{t}.\,y_{i}\,\left(\sum_{m=1}^{3}\beta_{m}\,{\left(w^{m}\right)}^{T}\,\phi^{m}\,\left(x_{i}^{m}\right)+b\right)\geq 1-\xi_{i}\\ \xi_{i}\geq 0,i=1,2,\ldots ,\text{n} \end{array}$$

where *n* denotes the sample size, $x_{i}^{1},x_{i}^{2}$ and ${\text{x}}_{i}^{3}$ represent the connection value, the global and nodal graph measurements of the *i*-th sample, and *y*_*i*_ ∈ {1,−1} correspond to its class label, respectively, and m denotes the corresponding index of modality,*w^m^* represents the normal vector of the hyperplane in the Hilbert kernel space (RHKS), ϕ*^m^* represents the mapping function from the original space to the present RHKS, and β_*m*_ denotes the weight of the m-th modality. Then, the dual form of the MK-SVM can be represented as:


(3) $$\begin{array}{c} \max_{\alpha }\sum_{i=1}^{n}\alpha_{i}-\frac{1}{2}\,\sum_{i,j}\alpha_{i}\,\alpha_{j}\,y_{i}\,y_{j}\,\sum_{m=1}^{3}\beta_{m}\,k^{m}\,\left(x_{i}^{m},y_{i}^{m}\right)\,\\ \text{s}.\text{t}.\sum_{\text{i}=1}^{\text{n}}\alpha_{i}\,y_{i}=0\\ 0\leq \alpha_{\text{i}}\leq C,i=1,2,\ldots ,\text{n} \end{array}$$

where $k^{m}\,\left(x_{i}^{m},y_{i}^{m}\right)=\phi^{m}\,{\left(x_{i}^{m}\right)}^{T}\,\phi^{m}\,\left(x_{j}^{m}\right)$ denotes a kernel matrix. Finally, the predictive level based on MK-SVM can be formulated as follows:


(4) $$f\,\left(x_{1},x_{2},\ldots ,x_{M}\right)=\text{sign}\,\left(\sum_{\text{i}=1}^{\text{n}}y_{i}\,\alpha_{i}\,\sum_{m=1}^{M}\beta_{m}\,k^{m}\,\left(x_{i}^{m},x^{m}\right)+b\right)$$

### Feature Selection and Validation

**FIGURE 1:**
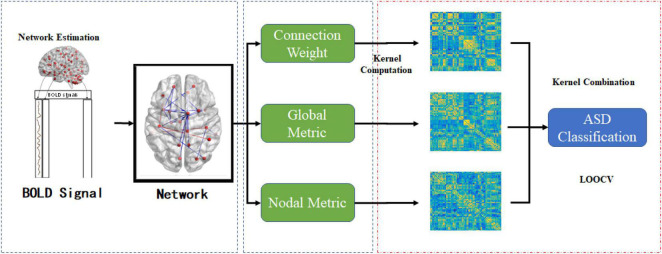
The entire pipeline of the proposed ASD classification task under the multiple graph view.

To alleviate the confounding effect of the different steps in the classification pipeline, we conducted the simplest feature selection method (i.e., *t*-test with *p* < 0.05). Additionally, to evaluate the performance of different classification methods, we adopted the most commonly used leave-one-out cross-validation (LOOCV) strategy owing to the limited sample size (Li et al., 2020a). Specifically, the optimal parameter (hyper-parameter C for MK-SVM) was selected in the inner cross-validation, and the classification performance was evaluated in the outlier validation loop. The range of the hyperparameter *C* was from  2^−5^ to 2^5^ . We compared the classification performance of a single aspect [i.e., connection weight (C), global typological information (G), nodal typological information (N)], and combinations of different modes (i.e., C + G, C + N, G + N, and C + G + N). The entire pipeline used in this study is shown in Figure 1. In contrast to the traditional methods which only utilize the information of connections, global graph metrics or nodal graph metrics, it should be noted that our novelty is that we using kernel combination trick and firstly combine the information from different modal for ASD identification.

### Classification Measurements

The classification performance of information combination methods is evaluated *via* several measurements, including sensitivity, specificity, and accuracy. The mathematical definitions of these three measures are as follows:


(5) $$A\,c\,c\,u\,r\,a\,c\,y=\frac{T\,r\,u\,e\,P\,o\,s\,t\,i\,v\,e+T\,r\,u\,e\,N\,e\,g\,a\,t\,i\,v\,e}{\begin{array}{c} T\,r\,u\,e\,P\,o\,s\,t\,i\,v\,e+F\,a\,l\,s\,e\,P\,o\,s\,t\,i\,v\,e+T\,r\,u\,e\,N\,e\,g\,a\,t\,i\,v\,e\\ +F\,a\,l\,s\,e\,N\,e\,g\,a\,t\,i\,v\,e \end{array}}$$

(6) $$S\,e\,n\,s\,i\,t\,i\,v\,i\,t\,y=\frac{T\,r\,u\,e\,P\,o\,s\,t\,i\,v\,e}{T\,r\,u\,e\,P\,o\,s\,t\,i\,v\,e+F\,a\,l\,s\,e\,N\,e\,g\,a\,t\,i\,v\,e}$$

(7) $$\text{S}\,p\,e\,c\,i\,f\,i\,c\,i\,t\,y=\frac{T\,r\,u\,e\,N\,e\,g\,a\,t\,i\,v\,e}{T\,r\,u\,e\,N\,e\,g\,a\,t\,i\,v\,e+F\,a\,l\,s\,e\,P\,o\,s\,t\,i\,v\,e}.$$

Additionally, the receiver operating characteristic curve (ROC) and AUC of these methods are also provided.

*A*_*r*_, assortativity; *E*_*global*_, global efficiency; *L*_*p*_, characteristic path length; Q, modularity score; *E*_*local*_, local efficiency; *C*_*p*_, clustering coefficient; *Hr*, hierarchy; σ, small-world; γ, normalized clustering coefficient; *S*_*r*_, synchronization; λ, normalized characteristic path length. *p-value < 0.05.

## Results

### Graph Theory Measurements of Functional Brain Connectome

The results of the graph measurements of the ASD and NC groups are shown in Table 3. The results illustrated that *Lp*, λ, *Ar*, and *Sr* were increased, whereas *C*_*p*_, γ, *E*_*global*_, and *Hr* were decreased in ASD. Statistical analyses revealed that γ was significantly decreased in the ASD group compared to the NC group (*p* < 0.05).

**TABLE 3:** Graph theory measurements of the functional brain connectome.

Graph theory measurements	ASD	NC
*C*_*p*_	0.2643 ± 0.01	0.2651 ± 0.01
*Lp*	0.8595 ± 0.05	0.8295 ± 0.04
γ*	1.0418 ± 0.10	1.0785 ± 0.07
λ	0.5002 ± 0.01	0.5001 ± 0.01
σ	0.9054 ± 0.09	0.9378 ± 0.06
*Q*	16.2912 ± 1.52	16.9097 ± 1.47
*E*_*global*_	0.2588 ± 0.01	0.2590 ± 0.01
*E*_*local*_	0.3439 ± 0.01	0.3549 ± 0.01
*Ar*	0.1647 ± 0.04	0.1510 ± 0.04
*Hr*	−0.0034 ± 0.04	0.0075 ± 0.04
*Sr*	−11.3854 ± 3.25	−12.2578 ± 3.44

*p value < 0.05.

### Degree Analysis of the Functional Brain Connectome

**FIGURE 2:**
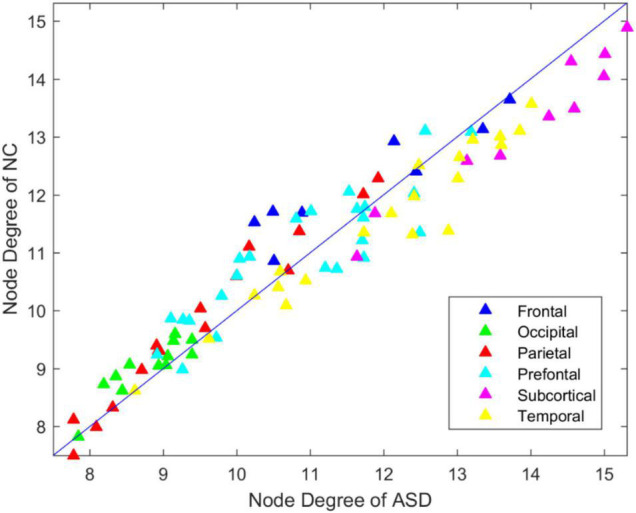
Degree distribution of ASD and NC group.

To investigate the degree distribution of the estimated brain network, we reported the mean degree of each node in the ASD and NC groups. As shown in Figure 2, the degree in the frontal, occipital parietal, and prefrontal regions tended to decrease in ASD, while it tended to increase in the temporal and subcortical regions. The 13 significant nodes with an average degree in the ASD and NC groups are listed in Table 4. The hub nodes of the ASD and NC groups are shown in Table 5. It was evident that most nodes overlapped across ASD and NC groups, including INS.L, PUT. L/R, PAL.L/R, INS.R, ROL.L, STG.L, ROL.R, AMYG.L/R, ACG.R, STG.R, and HES.L. Additionally, several specific hub nodes existed that corresponded to different groups. Specifically, in estimated brain network, ACG.L and IOG.R were only noted in the hub nodes of the NCs, while THA.L, HES.R, and HIP.L/R were only noted in ASD group as hub node.

**TABLE 4:** Significant nodes with the average degree in the ASD and NC groups.

Node	ASD	NC	*p*-Value
HIP.R	12.881 ± 1.93	11.382 ± 2.23	0.0009
PHG.R	12.391 ± 2.00	11.318 ± 1.87	0.0094
SMA.L	10.237 ± 2.50	11.528 ± 2.22	0.0104
PAL.L	14.594 ± 2.21	13.486 ± 1.98	0.0132
PCG.L	10.169 ± 2.08	11.101 ± 1.41	0.0135
OLF.R	12.494 ± 2.65	11.348 ± 1.71	0.0152
PUT.L	15.001 ± 1.97	14.044 ± 1.81	0.0175
SMA.R	10.494 ± 2.59	11.709 ± 2.32	0.0197
ORBsup.L	10.037 ± 2.04	10.893 ± 1.62	0.0283
THA.L	13.588 ± 2.15	12.676 ± 1.90	0.0341
SFGmed.R	10.809 ± 1.96	11.587 ± 1.57	0.0385
PAL.R	14.249 ± 2.23	13.353 ± 1.96	0.0432
SFGdor.R	9.103 ± 1.60	9.858 ± 1.93	0.0446

**TABLE 5:** Degree hubs of the MCI and NC groups based on the SR method.

AAL number	Corresponding brain region	Subnetwork
**ASD**		
29	Insula_L	Salience
74	Putamen_R	Subcortical
73	Putamen_L	Subcortical
75	Pallidum_L	Subcortical
30	Insula_R	Salience
76	Pallidum_R	Subcortical
81	Temporal_Sup_L	Ventral attention
42	Amygdala_R	Memory retrieval
17	Rolandic_Oper_L	Cingulo-opercular task Control
79	Heschl_L	Auditory
77	Thalamus_L	Subcortical
82	Temporal_Sup_R	Ventral attention
18	Rolandic_Oper_R	Auditory
41	Amygdala_L	Memory retrieval
32	Cingulum_Ant_R	Salience
78	Thalamus_R	Subcortical
80	Heschl_R	Auditory
37	Hippocampus_L	Default mode network
**NC**		
29	Insula_L	Salience
74	Putamen_R	Subcortical
30	Insula_R	Salience
73	Putamen_L	Subcortical
17	Rolandic_Oper_L	Cingulo-opercular task Control
81	Temporal_Sup_L	Ventral attention
75	Pallidum_L	Subcortical
76	Pallidum_R	Subcortical
18	Rolandic_Oper_R	Auditory
31	Cingulum_Ant_L	Default mode
42	Amygdala_R	Memory retrieval
32	Cingulum_Ant_R	Salience
82	Temporal_Sup_R	Ventral attention
41	Amygdala_L	Memory retrieval
33	Cingulum_Mid_L	Cingulo-opercular task Control
79	Heschl_L	Auditory
77	Thalamus_L	Subcortical

### Betweenness Analysis of the Functional Brain Connectome

**FIGURE 3:**
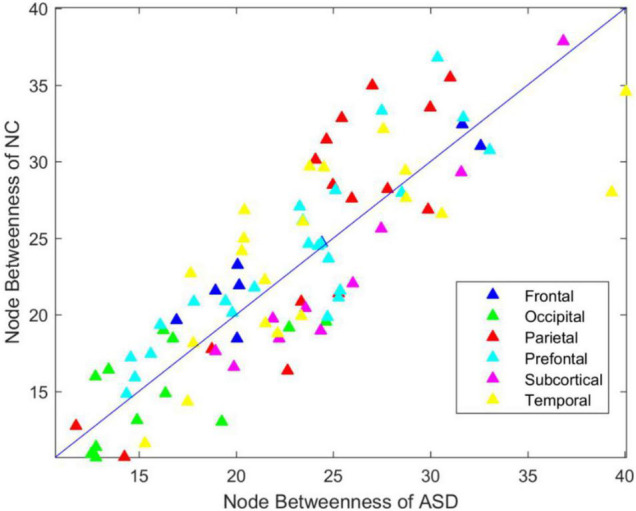
Betweenness distribution of ASD and NC group.

To investigate the betweenness distribution of the estimated functional brain connectome, we noted the mean betweenness of the ASD and NC groups (Figure 3). This finding illustrated that betweenness in the frontal, parietal, and prefrontal regions tended to decrease in ASD, while tended to increase in the subcortical regions. The significant nodes with average betweenness in the ASD and NC groups are listed in Table 6.

**TABLE 6:** Significant nodes with the average betweenness in the ASD and NC groups.

Node	ASD	NC	*p*-Value
IOG.L	19.257 ± 15.42	13.021 ± 8.82	0.0187
FFG.R	39.324 ± 27.00	28.005 ± 18.31	0.0203
PCG.L	25.433 ± 11.09	32.846 ± 18.29	0.0216
TPOsup.L	23.775 ± 11.54	29.680 ± 13.39	0.0261
THA.L	24.352 ± 13.45	18.941 ± 9.25	0.0264
AMYG.L	20.436 ± 11.77	26.824 ± 16.73	0.0305
PUT.L	26.008 ± 9.69	22.061 ± 8.17	0.0372
HES.R	15.299 ± 10.82	11.609 ± 5.98	0.0448
IPL.R	27.009 ± 15.37	34.970 ± 21.59	0.0454

The betweenness hub nodes of ASD patients and NCs are shown in Table 7. The results illustrated that most betweenness hubs overlapped across ASD and NC groups, including FFG.L, INS.L, ACG.L/R, DCG.L/R, IFGoperc.R, PCUN.R, IPL.L, and SPG.L. The FFG.R, HIP.L, and INS.R were only noted in hub nodes of the NCs, while IPL.R, ORBsupmed.L, PCG.L, PHG.R, SPG.R, PCG.R, and TPOsup.L were only noted in ASD group as hub node.

**TABLE 7:** Betweenness hubs of the MCI and NC groups based on the SR method.

AAL number	Corresponding brain region	Subnetwork
**ASD**		
55	Fusiform_L	Default mode network
56	Fusiform_R	Default mode network
29	Insula_L	Salience
32	Cingulum_Ant_R	Salience
34	Cingulum_Mid_R	Salience
12	Frontal_Inf_Oper_R	Frontoparietal task control
33	Cingulum_Mid_L	Default mode network
30	Insula_R	Salience
68	Precuneus_R	Default mode network
37	Hippocampus_L	Default mode network
31	Cingulum_Ant_L	Default mode network
61	Parietal_Inf_L	Default mode network
59	Parietal_Sup_L	Fronto-parietal task control
**NC**		
29	Insula_L	Salience
31	Cingulum_Ant_L	Default mode network
68	Precuneus_R	Default mode network
62	Parietal_Inf_R	Default mode network
55	Fusiform_L	Default mode network
61	Parietal_Inf_L	Default mode network
25	Frontal_Mid_Orb_L	Default mode network
12	Frontal_Inf_Oper_R	Frontoparietal task control
35	Cingulum_Post_L	Memory retrieval
33	Cingulum_Mid_L	Default mode network
40	ParaHippocampal_R	Default mode network
60	Parietal_Sup_R	Dorsal attention
34	Cingulum_Mid_R	Default mode network
32	Cingulum_Ant_R	Salience
36	Cingulum_Post_R	Default mode network
83	Temporal_Pole_Sup_L	Cingulo-opercular task control

### Consensus Connections Analysis

**FIGURE 4:**
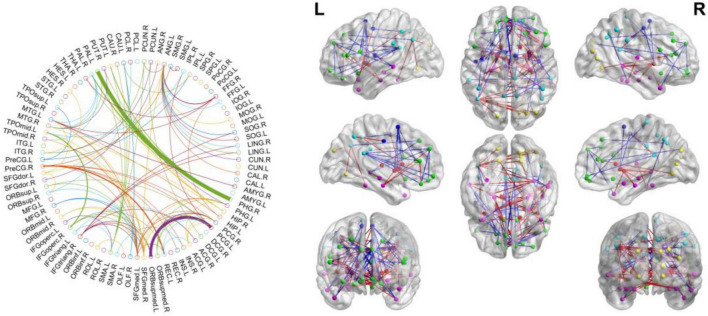
Consensus connection over the LOOCV by *p*-value < 0.05. The red line in the right figure represents the weights in ASD tends to be increased while the blue line represents decrease.

We noted all selected connections during the entire validation process, that is, consensus connections, as shown in Figure 4 because the selected connections in each inner validation loop could be different. Specifically, we selected the connections with a p-value < 0.05, in each loop to train the classifier, which resulted in the obtainment of 103 consensus connections. The most significant connection in consensus connection was PHG.R–PAL.L. The red line in the right side of figure represents the weights in ASD, which tends to increase, while blue line represents a decrease.

### Classification

**FIGURE 5:**
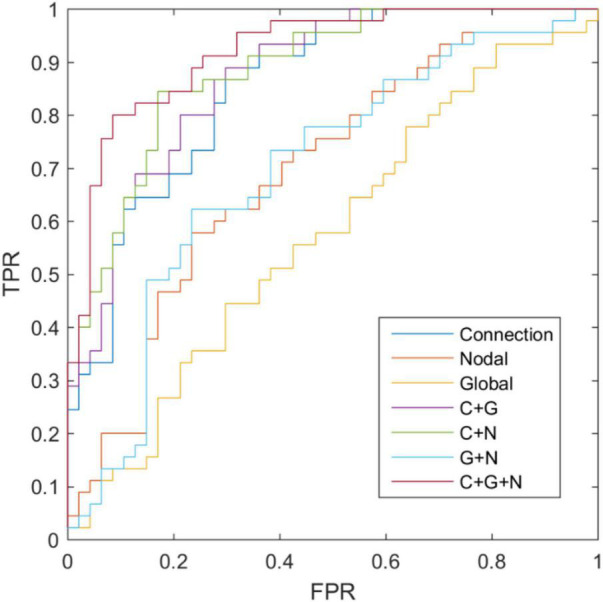
Receiver operating characteristic curve of classification based on different connectome features.

To confirm the information combination trick, we also validated the performance of the single kernel SVM classification result based on the connection, global measurements, and nodal measurements, and the results have been depicted in Table 8 and Figure 5. The classification accuracy of connection weight, global graph measurements, and nodal graph measurements were estimated to be 72.82, 63.04, and 54.34%, respectively. Additionally, using DeLong’s non-parametric statistical significance test (Zhang et al., 2016), C + G + N methods have been found to be significantly superior to Connection, Nodal, Global, C + G, C + N, and G + N methods under 95% confidence intervals with *p*-values of 0.004, 5×10^−6^, 3×10^−10^, 0.0247, 0.0278, and 1×10^−5^, respectively. These results revealed the superiority of the information combination strategy.

**TABLE 8:** Classification performance corresponding to different methods.

Method	Accuracy	Sensitivity	Specificity	AUC
Connection (C)	72.82	73.33	72.34	0.8539
Nodal (N)	63.04	66.67	59.57	0.6921
Global (G)	54.34	57.78	51.06	0.5726
C + G	76.09	80.00	72.34	0.8728
C + N	79.34	**84.44**	74.46	0.8841
G + N	67.39	73.33	61.70	0.6950
C + G + N	**82.60**	**84.44**	**80.85**	**0.9191**

*Boldface denotes the best performance for each column.*

## Discussion

In the present study, we aimed to explore the biomarkers and pathological mechanisms of brain network connectivity in individuals with ASD. Our results indicated that the combination of three modalities (i.e., connection weight, global measurements, and nodal measurements) using MK-SVM could significantly improve the classification performance of ASD individuals, since such methods not only utilized the information of the connection weight but also effectively incorporated several topological inputs. Moreover, the distribution of the discriminative brain regions and altered patterns of the brain connectome revealed the pathological mechanisms of individuals with ASD.

### Distribution of the Discriminative Brain Regions and Brain Networks

Regarding functional connectivity at the whole brain level, we found that the consensus connection with the most significant difference between ASD and NC was PHG.R–PAL.L. Additionally, the brain regions with significant differences in degree and betweenness between the ASD and NC groups were mainly distributed in the medial temporal lobe (e.g. HIP, PHG, and PCG), subcutaneous nuclei (e.g. PUT, PAL, and THA), and frontal and occipital parietal lobes. Meanwhile, the hub nodes of the ASD and NC groups, defined by degree and betweenness, mostly overlapped with the above-mentioned brain regions. Among them, brain regions, such as HIP, PHG, AMYG, and CG, belong to the limbic system. Therefore, our results indicated that the discriminative brain regions of ASD patients were mainly distributed in the limbic system, subcutaneous nuclei, cortex, and connections among them. Furthermore, by projecting these brain regions into brain subnetworks, we found that most were involved in the DMN, SN, attention network, frontoparietal network, and social network (Schmälzle et al., 2017). These sub-networks play an important role in learning and memory (Sanjeevan et al., 2020), emotional expression (Sagar-Ouriaghli et al., 2018), behavior control, and social skills (Sato and Uono, 2019).

### Altered Pattern of the Brain Network Connectome in Autism Spectrum Disorder

Graph theoretic analysis conducted in our study indicated that in terms of global graph measurements, *Lp*, λ, *Ar*, and *Sr* were increased, whereas *C*_*p*_, γ, σ, *Q*, *E*_*global*_, *E*_*local*_, and *Hr* were decreased in ASD. Although only γ seemed to be statistically significant, the graph theory attributes that reflected the segregation function of brain networks, including *C*_*p*_, γ, Q, and *E*_*local*_, exhibited a decreased trend in ASD patients. Meanwhile, the graph theory attributes that reflected the integration function of brain networks, such as *Lp* and λ, exhibited an increased trend in individuals with ASD. Therefore, our results suggested that patients with ASD exhibited abnormal functional connectivity. This finding was also mentioned in previous studies (Just et al., 2004; Kana et al., 2011; Zu et al., 2019). Our research further revealed that ASD patients showed enhanced integration function and weakened segregation functions of the brain network. This suggests that the ability to rapidly synthesize specialized information from distributed brain regions has increased, while the occurrence of specialized processing within densely interconnected groups of brain regions has decreased (Rubinov and Sporns, 2010).

Moreover, in terms of local graph measurements, we found that the topological attributes of ASD, such as degree, betweenness, and hub nodes, exhibited abnormalities in multiple brain areas in the cortex-subcortical circuits. Among them, the functional connections related to the medial temporal lobe (e.g. the hippocampus, parahippocampal gyrus, and entorhinal cortex) and subcutaneous nuclei (e.g. putamen and pallidum) were mainly increased, while the related connections of the frontal lobe, parietal lobe, and occipital lobe were mainly decreased. In previous studies, the dysfunction of connections has been demonstrated to be associated with abnormal social, language, and other behaviors of ASD patients (Gao et al., 2019; Verly et al., 2014; Sato and Uono, 2019). Additionally, the hub nodes of ASD and NCs mostly overlapped. Our results indicated the disappearance of certain hubs in the ASD group, which suggested that the brain network integration function of ASD might have changed. This might be related to the pathological changes in ASD.

### Classification of Multi-Kernel Support Vector Machine

The classification results illustrate that combination of more information can be considered to effectively enhance the classifier for ASD diagnosis. Moreover, although global theory achieves 54.24% accuracy, it can provide a 6.52% accuracy gain for C + N. The results indicate that different types of information can provide different types of discriminative information for ASD diagnosis, which further confirms the effectiveness of the proposed information combination method. More importantly, such results are achieved with the use of only single modal data, that is, fMRI, which thereby provides novel insights for improving the identification performance in such neurodevelopmental disorders.

## Conclusion

We report the application of FBNs as well as their topological information for investigation of potential biomarkers of ASD in the afflicted individuals. The discriminative connections of FBNs highlighted the abnormality of connections between the PHG.R and PAL.L of the ASD groups. Additionally, the combination of the information derived from the FBN with MK-SVM helped achieve the best classification performance and significantly outperformed the performance achieved using only single connection information. In the end, due to the limited sample sizes, it is still necessary for us to verify the robustness and generalization ability of the proposed methods on larger and higher quality databases in the future.

## Data Availability Statement

The original contributions presented in the study are included in the article/supplementary material, further inquiries can be directed to the corresponding authors.

## Author Contributions

XX and LP designed the study and drafted the manuscript. LP and XL collected the MRI data. XC and XX analyzed and interpreted the results of the data. XG and XC revised the manuscript. All authors approved the final manuscript.

## Conflict of Interest

The authors declare that the research was conducted in the absence of any commercial or financial relationships that could be construed as a potential conflict of interest.

## Publisher’s Note

All claims expressed in this article are solely those of the authors and do not necessarily represent those of their affiliated organizations, or those of the publisher, the editors and the reviewers. Any product that may be evaluated in this article, or claim that may be made by its manufacturer, is not guaranteed or endorsed by the publisher.

## Footnotes

1
https://www.fil.ion.ucl.ac.uk/spm

2
http://restfmri.net/forum/index.php

3
www.nitrc.org/projects/gretna/
